# Effect of Flow Rate on *In Vitro* Aerodynamic Performance of NEXThaler^®^ in Comparison with Diskus^®^ and Turbohaler^®^ Dry Powder Inhalers

**DOI:** 10.1089/jamp.2015.1220

**Published:** 2016-04-01

**Authors:** Francesca Buttini, Gaetano Brambilla, Diego Copelli, Viviana Sisti, Anna Giulia Balducci, Ruggero Bettini, Irene Pasquali

**Affiliations:** ^1^ Department of Pharmacy, University of Parma, Parma, Italy.; ^2^ Institute of Pharmaceutical Science, King's College London, London, United Kingdom.; ^3^ Chiesi Farmaceutici SpA, Parma, Italy.; ^4^ Interdepartmental Center, Biopharmanet-TEC, University of Parma, Parma, Italy.

**Keywords:** aerodynamic assessment, extra-fine particle mass, NGI flow rate, NEXThaler^®^, Diskus^®^, Turbohaler^®^

## Abstract

***Background:*** European and United States Pharmacopoeia compendial procedures for assessing the *in vitro* emitted dose and aerodynamic size distribution of a dry powder inhaler require that 4.0 L of air at a pressure drop of 4 kPa be drawn through the inhaler. However, the product performance should be investigated using conditions more representative of what is achievable by the patient population. This work compares the delivered dose and the drug deposition profile at different flow rates (30, 40, 60, and 90 L/min) of Foster NEXThaler^®^ (beclomethasone dipropionate/formoterol fumarate), Seretide^®^ Diskus^®^ (fluticasone propionate/salmeterol xinafoate), and Symbicort^®^ Turbohaler^®^ (budesonide/formoterol fumarate).

***Methods:*** The delivered dose uniformity was tested using a dose unit sampling apparatus (DUSA) at inhalation volumes either 2.0 or 4.0 L and flow rates 30, 40, 60, or 90 L/min. The aerodynamic assessment was carried out using a Next Generation Impactor by discharging each inhaler at 30, 40, 60, or 90 L/min for a time sufficient to obtain an air volume of 4 L.

***Results:*** Foster^®^ NEXThaler^®^ and Seretide^®^ Diskus^®^ showed a consistent dose delivery for both the drugs included in the formulation, independently of the applied flow rate. Contrary, Symbicort^®^ Turbohaler^®^ showed a high decrease of the emitted dose for both budesonide and formoterol fumarate when the device was operated at airflow rate lower that 60 L/min. The aerosolizing performance of NEXThaler^®^ and Diskus^®^ was unaffected by the flow rate applied. Turbohaler^®^ proved to be the inhaler most sensitive to changes in flow rate in terms of fine particle fraction (FPF) for both components. Among the combinations tested, Foster NEXThaler^®^ was the only one capable to deliver around 50% of extra-fine particles relative to delivered dose.

***Conclusions:*** NEXThaler^®^ and Diskus^®^ were substantially unaffected by flow rate through the inhaler in terms of both delivered dose and fine particle mass.

## Introduction

Drug delivery and lung intrinsic deposition from a dry powder inhaler (DPI) are influenced by the inspiratory effort produced by the patient, the resistance of the inhaler and by the formulation characteristics.^(1–3)^ The deaggregation of the powder formulation is driven by a turbulent energy (measured as pressure change) that is created inside the inhaler by the interaction between the patient's inhalation pattern and the resistance of the DPI.^(4–6)^ The acceleration of the flow has been shown to be critical for deaggregation of the formulation. It has been reported that the acceleration rates achieved through a DPI is related to clinical efficacy^(7)^ and it has been resulted being lower in children than in COPD patients, whereas adults with asthma produce the steepest rates.^(8)^ Therefore, the respiratory patient profile is an issue that must be addressed for an efficient DPI performance. The development of nonbreath-actuated DPIs is a strategy to overcome this matter, although these types of inhalers are nowadays still in their infancy from the market point of view.^(9)^

According to the Guideline on the Pharmaceutical Quality of Inhalation and Nasal Products,^(10)^ it is recommended that DPIs show consistent delivery performance across a specific range of flow rates/inspiratory effort, which should be representative of what is achievable by the intended patient population. Current European Pharmacopoeia (Ph. Eur.) and United States Pharmacopeia (USP) compendial procedures for assessing DPI drug delivery performance require that 4.0 L of air at a pressure drop of 4 kPa be drawn through the inhaler to quantify delivered dose uniformity and aerodynamic particle size distribution.^(10,11)^

By contrast, the FDA's Draft Guidance for Industry “Metered Dose Inhaler (MDI) and Dry Powder Inhaler (DPI) Drug Products” recommends that the inhalation volume be limited to 2.0 L for the delivered dose analysis, while, for the assessment of the aerodynamic particle size distribution, more consideration may need to be given to flow rate selection and duration, although, for routine testing, the same flow rate and duration should be used as for the delivered dose testing.^(12)^ In this respect, a study^(13)^ of the effects of sampling volume on particle size distribution of aerosols emitted form DPIs has been recently published. The determinations using Next Generation Impactor resulted influenced by sampling volumes <2 L and had consequently advocated the use of pharmacopoeial conditions for determination of aerodynamic particle size distribution.

According to the producer NEXThaler^®^, a breath-actuated reservoir DPI has been recently designed with the purpose to release a consistent full therapeutic dose independently of the flow rate achieved by the patient. The device comprises functional groups of components assembled together. The dosing mechanisms meter the drug from a reservoir and the counting mechanisms include the breath-actuated mechanism that activates the dosing group only above a certain air flow rate, allowing the dose to be taken. Hence, the dose counter decrements only after an effective release of the therapeutic dose.^(14,15)^

The purpose of the present study was to compare the deposition profile and the dose delivery at different flow rates of Foster NEXThaler^®^ (beclomethasone dipropionate/formoterol fumarate) with other two DPIs, namely Seretide^®^ Diskus^®^ and Symbicort^®^ Turbohaler^®^.

Foster and Seretide are carrier-based formulations in which drugs microparticles, micronized by jet milling, are blended with coarse lactose crystals. The carrier of the first product is constituted by coarse lactose (212–355 μm) blended with a micronized lactose/magnesium stearate mixture.^(16)^ Seretide contains just coarse lactose as adjuvant to improve dose uniformity by increasing the mass of powder for each dose.^(17)^ The Symbicort formulation consists of soft aggregates of co-micronized drugs formulated with a small amount of lactose as excipient (1–5 μm).^(18)^

Furthermore, in the light of discrepancy between the compendial and regulatory test condition outlined above, the effect of the difference in the two test inhalation volumes (2 and 4 L) on the delivered dose and aerodynamic performance from the NEXThaler, was evaluated.

## Material and Methods

### Materials

Chiesi Farmaceutici (Parma, IT) provided Foster NEXThaler, a new breath-actuated DPI containing beclomethasone dipropionate (BDP) and formoterol fumarate (FF) (label claim: 100+6 μg, dispersed in 10 mg of carrier). Samples of Seretide Diskus containing fluticasone propionate (FP) and salmeterol (as xinafoate) (SX) (label claim: 250+50 μg, lactose up to 12.5 mg/dose) and Symbicort Turbohaler containing budesonide (BD)+FF (label claim: 160+4.5 μg+lactose monohydrate 730 micrograms per dose) were obtained from a local pharmacy in Italy.

### HPLC quantification of salmeterol and fluticasone propionate

Starting from the analytical conditions reported in literature,^19^ the column length was decreased from 250 mm to 150 mm (Hypersil BDS C18, 5 μm, 150×4.6 mm, Thermo Scientific, MA, USA) in order to improve tailing factors of the two active ingredients. A Thermo Fisher HPLC system equipped with a P4000 pump and an AS3500 autosampler were employed (Thermo Fisher Scientific Inc., Waltham, MA, USA). A Thermo Scientific UV6000 PDA was set at 250 nm for SX and 238 nm for FP, while the retention time was 3.0 and 6.7 min for SX and FP, respectively. LOQ was about 0.043 μg/mL for salmeterol and 0.035 μg/mL for fluticasone propionate. The linearity of response of salmeterol and fluticasone propionate was evaluated on standard solutions in the concentration range 0.04–13 μg/mL and 0.04–90 μg/mL, respectively, with a correlation coefficient R^2^: ≥0.99 in both of the cases.

### HPLC quantification of formoterol fumarate and beclomethasone dipropionate

Chromatographic quantifications of formoterol fumarate and beclomethasone dipropionate were carried out using a liquid chromatography equipped with Thermo Fisher P4000 pump and Thermo Fisher AS3500 autosampler (Thermo Fisher Scientific Inc). A Thermo Scientific UV6000 PDA operating at 223 nm for FF and 238 nm for BDP was employed. The injection volume was set at 50 μL and a Sinergy Fusion column C18, 4 μm, 50×4.6 mm (Phenomenex, Castel Maggiore, Bologna, IT) was used as stationary phase.

A gradient programme using a solution containing a water phase of 0.02 M of NaH_2_PO_4_, adjusted at the pH of 3.0, and acetonitrile was employed in order to elute first FF and then BDP. The column temperature was maintained at 40°C and the flow rate was set at 1 mL/min. The retention time for was 3.1 and 10 min for FF and BDP, respectively. LOQ was about 0.019 μg/mL for FF and 0.049 μg/mL for BDP. The linearity of response of FF and BDP was evaluated on standard solutions in the concentration range 0.02–2.2 μg/mL and 0.05–36 μg/mL, respectively, with a correlation coefficient R^2^: ≥0.99 in both of the cases.

### HPLC quantification of formoterol fumarate and budesonide

Formoterol fumarate and budesonide contained in the Symbicort product were determined by reversed phase HPLC with UV detection using the same procedure described above for the determination of FF and BDP. The detection wavelength was set at 223 nm and 258 nm for formoterol fumarate and budesonide, respectively. A gradient programme using a solution containing a 0.02 M of NaH_2_PO_4_, adjusted to pH of 3.0, and acetonitrile was employed in order to elute first formoterol fumarate and then budesonide. The retention time for was 4.4 and 9.3 min for FF and BD, respectively. LOQ was about 0.009 μg/mL for FF and 0.047 μg/mL for BD. The linearity of response of FF and BD was evaluated on standard solutions in the concentration range 0.01–2.2 μg/mL and 0.05–71 μg/mL, respectively, with a correlation coefficient R^2^: ≥0.99 in both of the cases.

### DPI intrinsic resistance

The intrinsic resistance for the three types of inhalers was determined using a Dose Uniformity Sampling Apparatus (DUSA), a critical flow controller TPK, and a HCP5 vacuum pump (all from Copley Scientific Ltd, Nottingham, UK). The relationship between pressure drop and flow rate was measured and the specific resistance of the inhalers was calculated from the slope of the linear relationships between the square root of pressure drop and the volumetric flow.^(6)^ Two inhalers for each type of device were tested and the mean value reported.

### Cascade impactor analysis

#### Effect of flow rates

The *in vitro* aerodynamic assessment of the three DPI products was carried out using a next generation impactor, NGI (Copley Scientific Ltd) equipped with a pre-separator and a micro orifice collector (MOC), following the procedure detailed in the European and US Pharmacopoeia.^(10,11)^ The NGI was connected to a HCP5 vacuum pump (Copley Scientific Ltd). For each single experiment, three doses were discharged into the NGI at 30, 40, 60, and 90 L/min for a duration of time such as to obtain an air passing volume of 4 L. The details of the experimental plan performed are illustrated in Table 1. This protocol was applied to two distinct devices for each inhaler in order to take into account possible intra-batch variability (NEXThaler batch #B10814, Diskus batch #3983A, and Symbicort batch #NF3942).

**Table 1.** Experimental Plan of Doses Collected Inside the NGI for Each of the Two Devices Discharged

*Flow rate (L/min)*	*Actuation (Dose number #)*
30	18-19-20
	38-39-40
	58-59-60
40	13-14-15
	33-34-35
	53-54-55
60	8-9-10
	28-29-30
	48-49-50
90	3-4-5
	23-24-25
	43-44-45

The required flow rate was obtained by adjusting the critical flow valve, ensuring that critical flow conditions were always maintained (flow meter DFM2000 and Critical Flow Controller Model TPK, Copley Scientific Ltd). NGI stages were coated with Tween 20/ethanol, 2% w/v solution, in order to prevent particle bouncing inside the impactor. After the required actuations were performed into the NGI, the powder deposited on the different portions of the impactor was collected using a 40:60 (v/v) water:methanol mixture, filtered (PTFE 0.45 μm, Sartorius AG, Goettingen, Germany) and then, drugs quantified by HPLC.

#### Effect of inhalation volume

Fine particle masses and fractions <5 μm of drugs released by Foster^®^ NEXThaler were also assessed using inhalation volumes of 2.0 and 4.0 L, adjusting the flow rate through the inhaler at 55 L/min±5% to provide a 4.0 kPa pressure drop across the device (flow meter DFM2000 and Critical Flow Controller Model TPK, Copley Scientific Ltd). As a consequence, the aspiration time was set at 2.18 or 4.36 s, respectively. Three doses were actuated into either NGI or Andersen Cascade Impactor, ACI (Copley Scientific Ltd) for each experiment and the test was performed in triplicate.

#### Data analysis

Fine particle mass lower than 5 μm (FPM<5 μm), extrafine particle mass lower than 2 μm (EFPM<2 μm), fine particle fraction (FPF%), mass median aerodynamic diameter (MMAD) and geometric standard deviation (GSD) were determined from the analysis of the NGI/ACI data. The calculation of the aerodynamic parameters was performed by using CITDAS (Copley Inhaler Data Analysis Software, UK).

MMAD and GSD were calculated based upon the inverse normal of the cumulative percentage under the stated aerodynamic diameter versus the log of the effective cut-off diameter. Linear regression of the five data points closest to 50% of the cumulative particle mass that entered the impactor was performed to compute the MMAD and GSD. The cut-off diameter of NGI stages was calculated and corrected for the different flow rates according to USP. In all impactor tests conducted, the mass balance was within±15% of the labelled dose.

### Delivered dose uniformity

The delivered dose uniformity was tested using a dose unit sampling apparatus (DUSA) operating at pressure drop of 4 kPa for the duration of time to allow 4 L of air as specified in USP 36. Furthermore, additional tests were performed using inhalation volumes of either 2.0 or 4.0 L at flow rates of 30, 40, 60, and 90 L/min. In all the cases, dose collection was carried out under critical flow control conditions. Ten delivered dose measurements (five for each of the two devices comprised between dose number #61–120) at each condition were obtained by collecting a single actuation; active pharmaceutical ingredients (APIs) deposited in the sampling apparatus were quantitatively recovered with 40:60 (v/v) water:methanol mixture and quantified by HPLC.

A high-retention borosilicate glass microfiber filter (Whatman, 934-AH, Sigma Aldrich, Milan, IT) was inserted in the DUSA or cascade impactors to collect the micro or submicron fraction of aerosol. The flow rate of each experiment was adjusted after placing the filter in the system for the analysis.

## Results and Discussions

The main attribute that controls the operational flow rate of a powder inhaler is the device specific resistance. A plot of the square root of pressure drop versus flow rate for the three types of inhaler investigated in this study was constructed and the relationship between the two variables was linear, as already described for several types of inhalers.^(6)^ The specific internal flow resistances (slopes obtained from the plots) for each device was determined. The resistance of the NEXThaler was 0.036 √kPa /(L/min) corresponding to a flow rate of 55 L/min at 4 kPa, while the Diskus resistance was 0.030 √kPa / (L/min) corresponding to a flow rate of 67 L/min at 4 kPa and the Turbohaler exhibited a resistance of 0.035 √kPa /(L/min), corresponding to a flow rate of 57 L/min at 4 kPa. Diskus is a low resistance inhalation device, whereas NEXThaler and Turbohaler can be considered to be of medium resistance.^(6)^

Large intra- and inter-inhaler dose emission differences may result in asthma and COPD exacerbations.^(20)^ This also may occur because of the absence of immediate therapeutic feedback when inhaling corticosteroids. Therefore, it is important that a DPI should deliver a consistent dose irrespective of a patient's PIF flow rate.

**FIG. 1.**
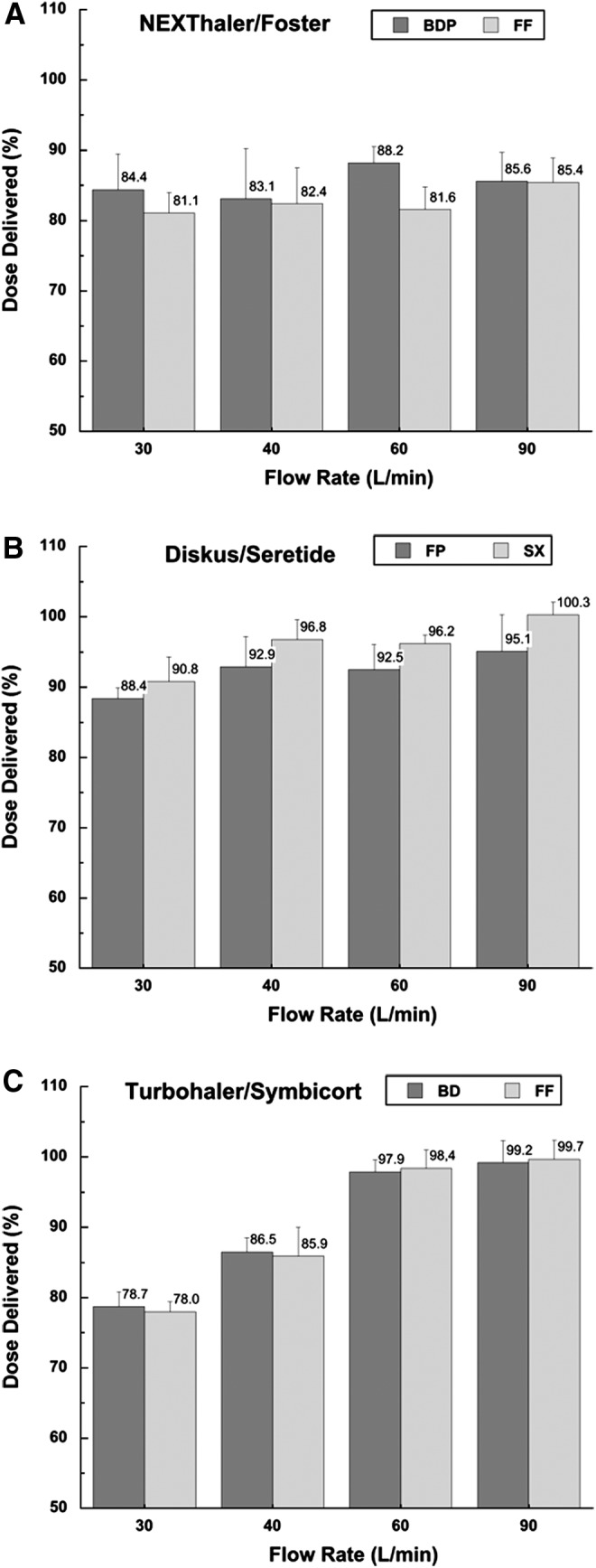
Delivered dose (% of the label claim) performance of the NEXThaler **(A)**, Diskus **(B)**, and Turbohaler **(C)** at different flow rates; inhalation volume 4L. Error bars represent standard deviations (*n*=6).

The peak inhalation flow (PIF) through the DPI usually influences the emitted dose, the aerosol size distribution, and the variability as well as lung deposition. Different PIF values can be reproduced *in vitro* through different pump flow rate. The emitted dose of the three devices was measured at different flow rates (30, 40, 60, and 90 L/min) in order to investigate the influence of this variable on the drug delivery. The delivered dose (DD) from the three inhalers, expressed as percentage of the label claim, is reported in Figure 1.

Foster^®^ NEXThaler^®^ showed a consistent dose delivery (81.1%–88.8%), for both the drugs included in the formulation, independently of the applied flow rate (*t*-test, *p*>0.05) in the range of 30–90 L/min. Similarly, Diskus^®^ did not show any significant decrease in the DD with decreasing flow rate (*t*-test, *p*>0.05). In this case all the values recorded were included in the range 88.4%–100.3% of the theoretical delivered dose.

Hence, the acceptance test criteria for these two devices were satisfied since no less than nine of the ten doses were in the range 75%–125% of the specified target-delivered dose and none outside the range 65%–135%. These results indicate that these two products provide the therapeutic dose in a wide inspiratory flow range.

Conversely, Turbohaler showed a high decrease of the emitted dose for both budesonide and formoterol fumarate when the device was operated at an airflow rate lower that 60 L/min (*p*<0.05). In particular, from 60 to 30 L/min the emitted dose of budesonide decreased from 97.9% to 78.7% with similar decrease in formoterol fumarate. The observed dependence of emitted dose of this inhaler on air flow velocity has been already demonstrated in previous studies.^(21,22)^ However, the dose released in this study at 30 L/min from Turbohaler was found to be significantly higher than the one around 37.5% both for FF and BD reported by Hill and Slater.^(23)^

Furthermore, it has been reported that Turbohaler functioned optimally *in vivo* only at high inspiratory flow rates.^(24)^ Average total lung deposition of terbutaline sulfate or budesonide via Turbohaler in healthy volunteers ranged from 21%–32% of the dose when a normal inhalation flow rate (60 L/min) was used. When the inspiration was conducted at a low rate (30 L/min) only a mean 15% of the dose was deposited in the lungs.

The dependence of the lung deposition on the inhalation flow rate was a feature common to all the first marketed dry powder inhalers. The advances in this field pushed scientists to address this huge issue and to develop inhalers able to release the required drug amount, also in case of patients such as children who do not achieve the recommend PIF through their prescribed DPI.

**FIG. 2.**
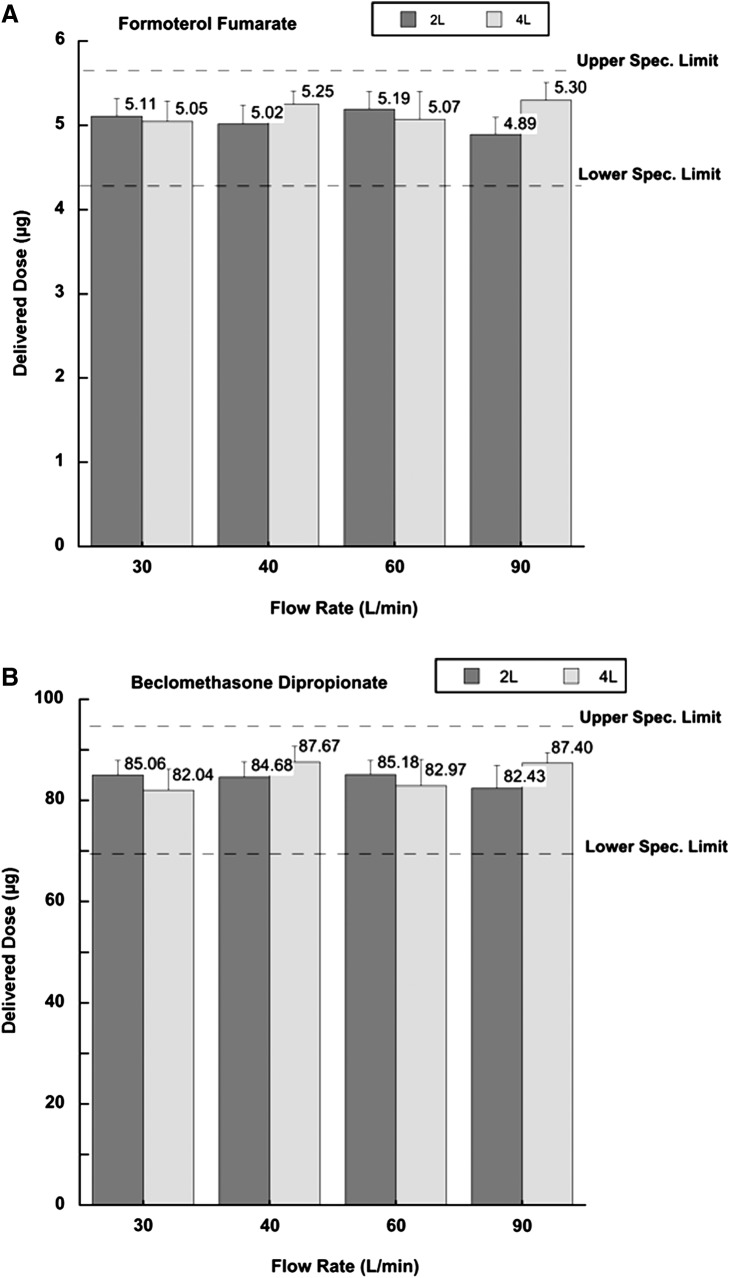
Delivered dose of formoterol fumarate **(A)** and beclomethasone dipropionate **(B)** from NEXThaler as a function of inhalation volume (2L and 4L) and different air flow rates (values are mean (SD), *n*=10).

A further test was performed on NEXThaler in order to evaluate whether the difference in the inhalation volumes might impact on the delivered dose. Figure 2 shows the delivered dose of a single actuation of FF and BDP when the inhaler was passed by 2 L or 4 L of air at different flow rate (30, 40, 60, and 90 L/min).

The delivered doses, express as μg of FF and BDP, across the flow rate range 30–90 L/min were not significantly affected by the different inhalation volumes and remained with a variation largely within±15% of the specification value. The values measured were around 5 μg for formoterol fumarate and 85 μg for beclomethasone dipropionate, respectively (Fig. 2).

These results demonstrate the capability of NEXThaler to release the labelled dose independently of the rate and amount of air employed to extract the dose. The dose is released in the first second after the activation of the device and does not required a huge amount of air to be extracted.^(25)^ This is mainly attributable to the intrinsic presence of a releasing breath actuated mechanism, as further discussed, which could facilitate an appropriate use also in young children or elderly people. Inhalation maneuvers are crucial for achieving the therapeutic effect, but, disappointingly, these are difficult to fulfil in specific patient groups, such as elderly patients and children.^(26)^

Beside the emitted dose, the main parameter that affects the clinical efficacy of an inhaler is the fine particle mass (i.e., the dose of active compound), with an aerodynamic size lower than 5 μm, able to penetrate and deposit into the lung. In the case of a DPI product, the fine particle mass is considerably affected both by the inhaler characteristics and by the inhalation profile since the formulation is extracted, de-aggregated, and dispersed by the patient inhalation maneuver. Furthermore, the importance to distribute the drugs throughout the whole lungs, including the small airways, to control asthma and COPD symptoms has been recently reported.^(27,28)^

**FIG. 3.**
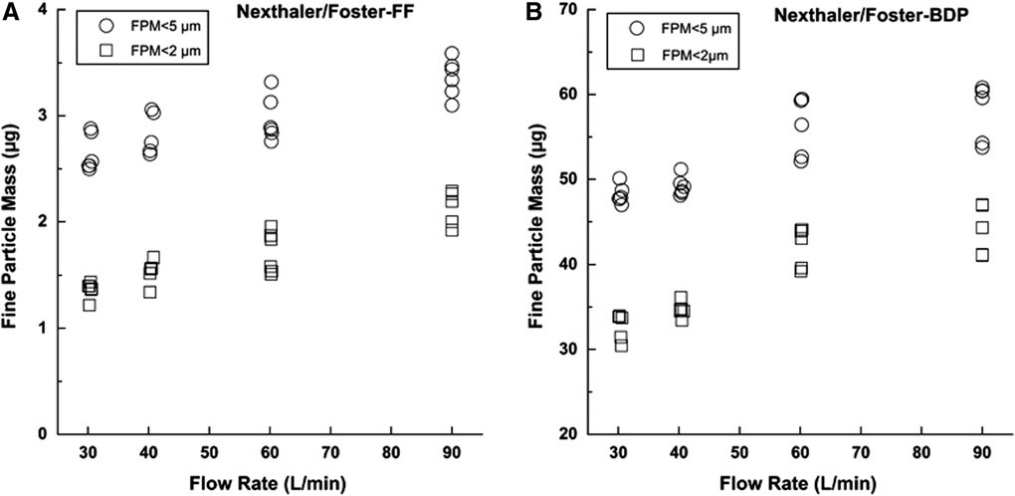
Fine particle mass expressed as amount of formoterol (FF) **(A)** and beclometasone dipropionate (BDP) **(B)** below 2 μm and below 5 μm when NEXThaler was activated at different flow rates, inhalation volume 4L (*n*=6).

**FIG. 4.**
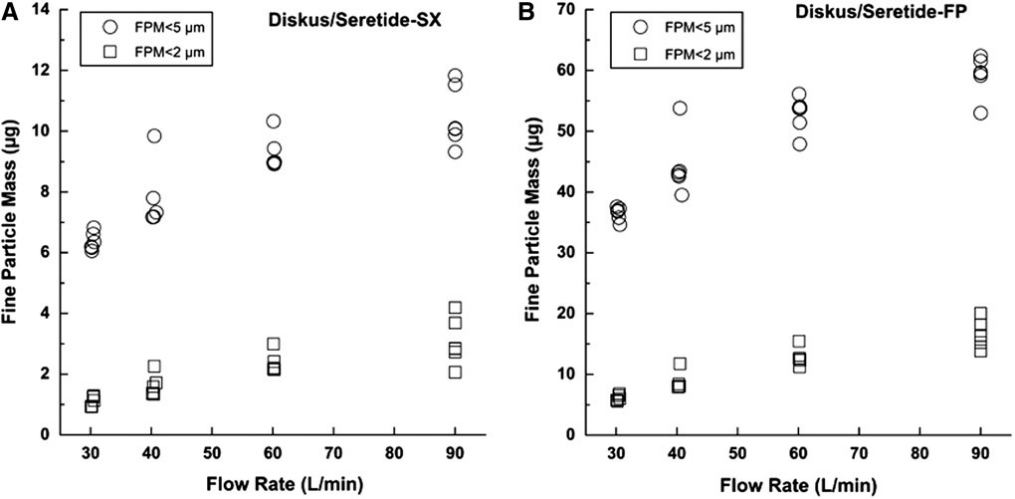
Fine particle mass expressed as amount of salmeterol (as xinafoate) (SX) **(A)** and fluticasone propionate (FP) **(B)** below 2 μm and below 5 μm when Diskus was activated at different flow rates, inhalation volume 4L (*n*=6).

**FIG. 5.**
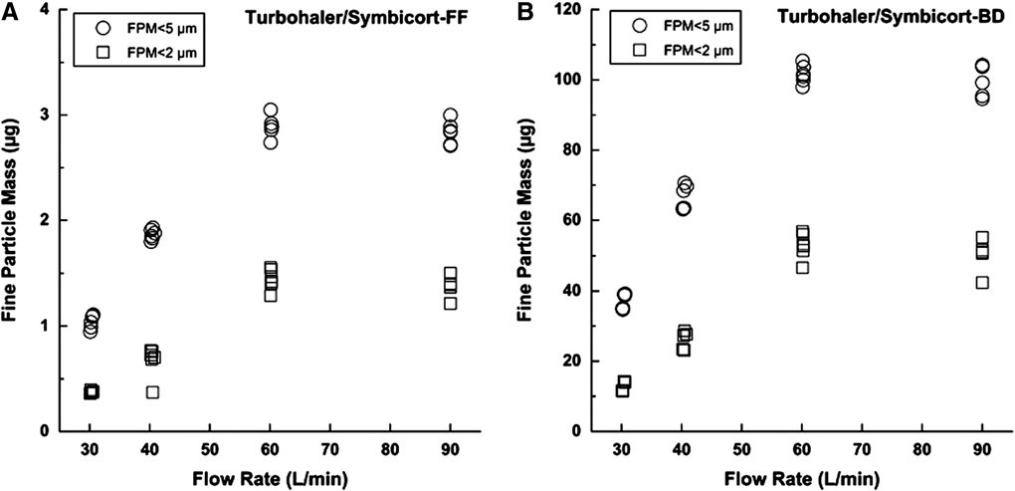
Fine particle mass expressed as amount of formoterol (FF) **(A)** and budesonide (BD) **(B)** below 2 μm and below 5 μm when Turbohaler was activated at different flow rates, inhalation volume 4L (*n*=6).

For this reason, the extrafine particle mass (EFPM) (i.e., particle having size smaller than 2 μm) was in this work determined as well. Fine particle mass (FPM) below 5 μm and extrafine particle mass (EFPM) below 2 μm obtained upon the actuation of the three different inhalers at different flow rates are illustrated in Figure 3, Figure 4, and Figure 5. The values of delivered dose (DD), fine particle mass (FPM), and fine particle fraction (FPF) performed in triplicate on two devices of each type of inhaler at different flow rate are reported in Table 2. Mean values and standard deviations (reported into the brackets) are referred to the six tests performed at each flow rate.

**Table 2.** Delivered Dose, Fine Particle Mass, and Fine Particle Fraction Values of Drug Microparticles Released by Combination Product Foster^®^ NEXThaler^®^, Seretide^®^ Diskus^®^ and Symbicort^®^ Turbohaler^®^

*Flow (L/min)*	*DD (μg)*		*FPM (μg)*		*FPF (%DD)*	
*Foster^®^ NEXThaler^®^*						
	*BDP*	*FF*	*BDP*	*FF*	*BDP*	*FF*
30	84.4 (5.1)	4.9 (0.2)	48.2 (1.1)	2.6 (0.2)	57.3 (3.7)	54.5 (4.5)
40	83.1 (7.1)	4.9 (0.3)	49.2 (1.1)	2.9 (0.2)	59.5 (4.6)	57.9 (5.5)
60	88.0 (2.3)	4.8 (0.2)	58.4 (4.1)	3.4 (0.2)	66.3 (1.0)	64.9 (2.0)
90	85.6 (4.1)	5.1 (0.2)	57.9 (3.3)	3.4 (0.2)	67.6 (3.0)	65.6 (2.3)
*Seretide^®^ Diskus^®^*						
	*SX*	*FP*	*SX*	*FP*	*SX*	*FP*
30	45.4 (1.7)	220.9 (3.8)	6.4 (0.3)	36.5 (1.1)	14.0 (0.4)	16.5 (0.6)
40	48.4 (1.4)	232.3 (10.6)	7.7 (1.0)	44.3 (4.9)	16.0 (1.7)	19.1 (2.0)
60	48.1 (0.6)	231.2 (9.1)	9.3 (0.6)	52.9 (2.9)	19.3 (0.9)	22.9 (1.2)
90	50.2 (0.9)	237.6 (13.1)	10.5 (1.0)	59.2 (3.3)	20.8 (1.7)	25.0 (1.6)
*Symbicort^®^ Turbohaler^®^*						
	*BD*	*FF*	*BD*	*FF*	*BD*	*FF*
30	125.8 (3.3)	3.5 (0.1)	37.0 (2.2)	1.0 (0.1)	29.5 (2.5)	29.8 (1.8)
40	138.4 (3.2)	3.9 (0.2)	66.5 (3.5)	1.9 (0.1)	48.1 (3.4)	48.4 (2.9)
60	164.0 (3.3)	4.6 (0.1)	102.1 (2.9)	2.9 (0.1)	62.3 (2.7)	62.6 (1.4)
90	158.6 (4.9)	4.5 (0.1)	100.2 (4.4)	2.8 (0.1)	63.2 (2.6)	62.7 (3.0)

BD, budesonide; BPD, beclomethasone dipropionate; DD, delivered dose; FF, formoterol fumarate; FP, fluticasone proprionate; FPF, fine particle fraction; FPM, fine particle mass; SX, salmeterol xinafoate. Values are mean (SD) (*n*=6).

A statistical evaluation of results was performed by carrying out a *t*-test on the data obtained at different flow rates in comparison to those found at reference flow rate corresponding to 4 kPa of drop pressure (60 L/min). Although the dispersion data were obtained from two devices belonging to the same batch, so not representative of batch to batch variation, the analysis provide an accurate picture of the intra-batch dispersion. NEXThaler^®^ shows a consistent fine particle fraction (FPF) delivery independent of the applied flow rate (*t-*test, *p*>0.05); the only statistically significant difference observed was for the BDP FPM at 30 L/min (48.2 μg) that was lower from that obtained at 60 L/min (58.4 μg) (*t*-test, *p*<0.05) (Table 2).

According to the producer, the observed efficient performance of this device should be attributed to the presence of a release mechanism activated by the inspiration of the patient (Breath Activated Mechanism—BAM) that avoids the uncontrolled administration of the drug (dose protector). The device allows releasing the drug only when the inhalation of the patient reaches the minimum flow rate that ensures the activation of the BAM and the drug to reach the lungs in an effective manner, independent of the flow rate applied.^(14,29)^

In this respect, an additional investigation about manually or breath activated doses was performed and it was found that a NEXThaler device without BAM, releasing the drug at the start of the inspiration act, gave FPF values about 30% lower than those obtained with the breath activated NEXThaler (data not shown). This different behavior is explained by taking into account that dose release and the degree of deaggregation obtained from the initial part of the inhalation act are affected by the flow acceleration.^(25,29)^ The presence of BAM implies that the powder is released only when a specific threshold of inspiration flow rate value is attained (35 L/min).^(29)^ This effect results in an instantaneous release of the dose that occurs in 0.35 s,^(29)^ thus making irrelevant the following part of the patient inspiration pattern (peak value).

Diskus^®^ showed a significant decrease of the fine particle fraction (FPF) of salmeterol at 30 L/min, which resulted significantly different (about 40%) from the value obtained at 60 L/min (t-test, *p*<0.05). No other significant difference in FPF was observed for Diskus at the other flow rates. These data are in agreement with the report of Nielsen et al. who attested that the aerosolizing property of the Diskus in children was independent from the flow performance within the flow range of 30–90 L/min, which is the range relevant to 3–10-year-old children.^(30)^

Taking in consideration the formulation characteristics above reported, it is possible to conclude that an air flow rate higher than 30 L/min is adequate to cause a reliable detachment of drug microparticles form the carrier surface. Furthermore, it must be underline the presence of the lubricant magnesium stearate in the Foster formulation represents an additional feature helping the product performance. The intrinsic presence also of a fine lactose fraction inside the carrier is known to help the deaggregation of the drug microparticles.^(31)^

On the other hand, Turbohaler^®^ proved to be the inhaler most sensitive to changes in flow rate between 30 and 60 L/min (*t*-test, *p*<0.05), in terms of fine particle fraction (FPF) for both components. Symbicort showed a fine particle mass equal to 2.9 μg and 101.6 μg for FF and BD when the device was activated at 60 L/min. These values fall down to 1.0 and 37.0 μg, respectively, when the flow was decreased to 30 L/min (Fig. 5). These data show that a low flow rate cannot generate a suitable energy to finely de-aggregate the drug–lactose soft pellets released by this inhaler. However, it must be taken into consideration that the data collected at 30 L/min have a lower relevance considering that the pressure drops typically attained by the patient population is in the range 2–6 kPa a corresponding to a flow rate between 40–70 L/min through this inhaler; thus this is the most important range to be considered.

It has been shown that flow-dependent dose and fine particle mass emission have significant clinical implications.^(32)^ Turbohaler showed a reduced clinical efficiency at low inspiratory flow due to reduced efficiency of drug dispersion and emptying of the device. It was reported that lung deposition of terbutaline sulfate through Turbohaler was different at decreasing peak inspiratory flows.^(32)^ A study conducted in 10 asthmatic subjects showed a trend towards a reduced bronchodilator response at the lower flow rate, even if this did not reach statistical significance.

The authors explain this effect considering that increasing the flow rate through Turbohaler a reduced particle impaction in the oropharynx and an improved drug delivery to the lungs occurs.

For passive DPI, dispersion and deposition forces are controlled with the same inhalation maneuver. Deposition modeling and *in vivo* studies have shown that deposition in the lungs shifts to larger airways when the flow rate is increased.^(33,34)^ The greater the inspiratory flow, the greater the particle inertia, and thus the greater the fraction of particle deposited in the large airways. Interestingly, oropharyngeal deposition during fast inhalation (>60 L/min) increased particularly for 3- and 6- μm particles with a corresponding decrease in their total lung deposition and a consequent fall in FEV1 compared to 1.5 μm particles, less prone to inertial impaction and whose deposition is less dependent on inhalation technique.^(34)^

For this reason, when the aerosol is mainly composed by particles in the 3–6 μm range, the increase in the flow rate, that would result in an more effective dispersion, would be advantageous in order to compensate at least partly for a shift in deposition to higher airways and to make the lung deposition less variable. This behavior, observed for Turbohaler in this study, was also shown in a study conducted with Novolizer where the dispersion behavior overcompensated the shift in deposition and a highest lung deposition was obtained at the highest flow rate (90 L/min).^(35)^ On the other hand, when the aerosol is mainly composed of extrafine particles, the difference between *in vitro* aerosolization and *in vivo* lung deposition varying the flow is less pronounced,^(34)^ thus making the flow independent behavior a valuable feature that can guarantee a more controlled deposition in patients with different inspiratory flow rate.

A further characteristic of the dry powder formulation that can be calculated from data reported in Figures 3 to 5 is the ratio between extrafine particle mass and FPM: Foster provided higher values relative to other products. Both in the case of FF and BDP, the percentage of particle having mass smaller than 2 μm attained a value higher than 50% (reaching 80%) of that relevant to FPM <5 μm.

**FIG. 6.**
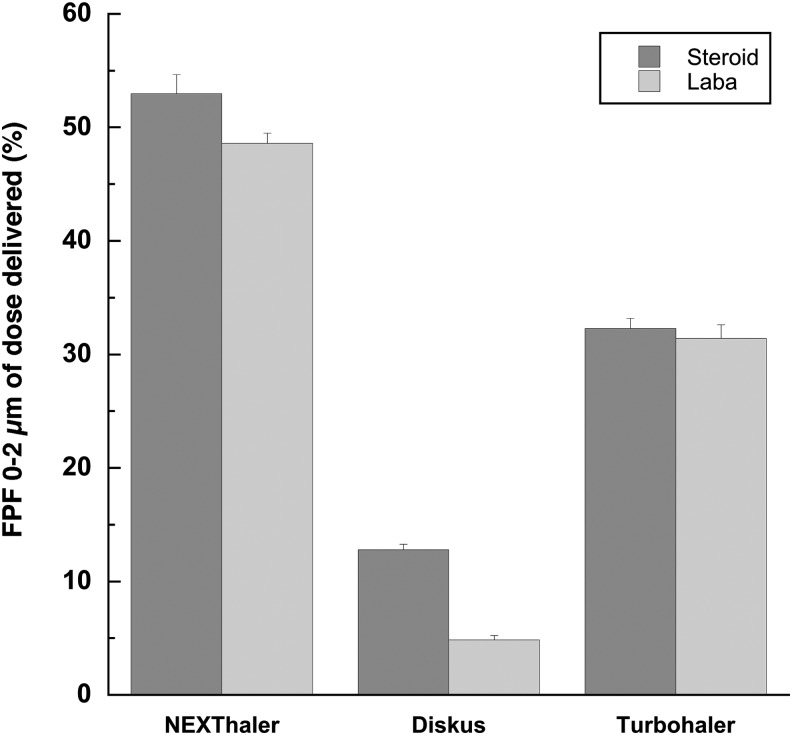
Extrafine particle fraction (0–2 μm) expressed as percentage of dose delivered of the long-acting beta agonist (LABA) and corticosteroid released from products at 60 L/min, inhalation volume 4L (*n*=6).

Seretide and Symbicort showed a lower release of extrafine mass (percentage value ranging between 25%–48% for Symbicort and 17%–27% for Seretide). In this respect, Figure 6 shows the FPF of particle size in the range 0–2 μm as percentage of dose delivered of the steroid (ICS) and long-acting beta agonist (LABA) when released at 60 L/min. Foster NEXThaler is the only steroid-LABA combination, among the combinations tested, capable to deliver around 50% of extrafine particles. The result is in agreement with the low MMAD value of this formulation microparticles, measured to be 1.1–1.7 μm, for BDP and FF, respectively, which let particles depositing *in vivo* in the peripheral lung.^(36)^ FPF of particle size in the range 0–2 μm for Diskus and Symbicort was around 10% and 30%, respectively.

This point is of particular importance as it has been demonstrated *in vivo* that, using a series of drug microparticle with different size range, smaller particles (1.5 μm) achieved greater total lung and more peripheral deposition.^(34)^ A scintigraphic study confirmed a high lung deposition (56% of the emitted dose in asthma patients) and uniform distribution throughout the entire bronchial tree of extrafine BDP/FF (400/24 μg) after administration through the NEXThaler dry powder inhaler.^(27)^

Although the study by Usmani et al. indicates that the highest clinical effect was not obtained with the smallest particles,^(34)^ other studies of extrafine inhaled corticosteroids (ICS) demonstrated larger improvements in functional and inflammatory parameters related to small airways abnormalities compared with non-extrafine formulations, even at lower extrafine ICS doses.^(37,38)^

Considering that for particles lower than 1 μm, sedimentation takes significantly longer compared to bigger particles, the issue of drug fraction exhaled should be taken into consideration. Interestingly, the study by Usmani et al. shows that for 1.5 μm particles, the fraction exhaled was already 21.9%, versus 8.3% and 2.3% for particles of 3 and 6 μm, respectively. Since higher lung deposition was observed with extrafine particles, apparently some mechanism preventing high exhaled drug fractions may be envisaged.

A further study reported that the extrafine beclomethasone–formoterol combination was not inferior to the equipotent doses of non-extrafine fluticasone–salmeterol in terms of efficacy and tolerability, with the advantage of a faster onset of bronchodilation.^(39–41)^ Nevertheless, the beclomethasone–formoterol combination demonstrated a greater and more specific effect on variables directly related to small airways function (i.e., the improvement of airway closing capacity).^(29,37)^ Although it cannot be excluded that the demonstrated effect may be attributed to other aspect related to the intrinsic pharmacological characteristics of the molecules compared, it is worthy underlining that in all the literature cases the effect of the particle size is investigated clinically by comparing equipotent doses of different drugs.

Furthermore, a recent study conducted using a Functional Respiratory Imaging demonstrated patients previously treated with ICS remained stable despite the lower dose, while ICS naive patients improved in terms of lobar hyperinflation when switching to an extrafine particle BDP/F formulation.^(42)^

**FIG. 7.**
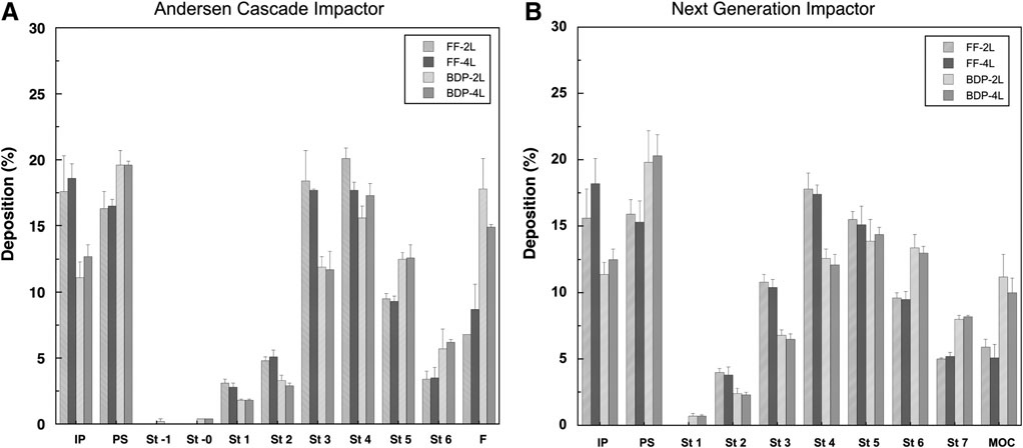
Stage by stage deposition profiles of formoterol fumarate **(A)** and beclomethasone dipropionate **(B)** inside the Andersen Cascade Impactor and Next Generation Impactor after NEXThaler aerosolization at 60 L/min, inhalation volume 2 and 4L (values are mean (SD), *n*=3).

Finally, the effects of sampling volume on particle size distribution of aerosols emitted from DPIs, was investigated using both ACI and NGI in order to assess the influence of air volume on the respirability of Foster product. Figure 7a and Figure 7b show the stage-by-stage deposition profiles in the ACI and NGI for BDP and FF at different sampling volume (2 and 4 L), expressed as percentage of delivered dose. The derived values of fine particle mass (FPM) and fine particle fraction (FPF), performed in triplicate, are reported in Table 3.

**Table 3.** Fine Particle Mass and Fine Particle Fraction Values of Beclomethasone Dipropionate and Formoterol Fumarate Aerosolized by Foster^®^ NEXThaler^®^ inside the Andersen Cascade Impactor and Next Generation Impactor at 2 or 4 L of Air through the Inhaler

*Formoterol fumarate*		*FPM (μg)*	*FPF (%)*
ACI	2L	3.1 (0.1)	65.1 (2.5)
	4L	3.2 (1.3)	64.1 (1.0)
NGI	2L	3.4 (0.2)	64.9 (2.0)
	4L	3.4 (0.1)	66.8 (0.8)

*Beclomethasone dipropionate*		*FPM (μg)*	*FPF (%)*
ACI	2L	57.4 (2.3)	66.3 (0.6)
	4L	61.2 (0.3)	67.7 (0.1)
NGI	2L	58.5 (2.4)	64.7 (0.3)
	4L	58.4 (4.1)	66.3 (1.0)

ACI, Anderson cascade impactor; FPF, fine particle fraction; FPM, fine particle mass; NGI, Next generation impactor.

As can be seen from the similarity of the stage-by-stage deposition profiles, the aerodynamic particle size distribution was unaffected by the inhalation volume for both drugs. These results can be explained by considering the previously cited publication by Corradi et al.^(29)^ showing that the metered powder dose is completely de-aggregated and delivered in 0.35 s, namely with 0.35 L of air at a flow rate of 60 L/min. Finally, it is worth noting that both drugs were co-deposited on the various stages of impactors, thus supporting the rationale development of the combination.

## Conclusions

This work demonstrates the importance of performing aerosol characterization tests not only following the quality requirement of USP and Eur Pharm, but also modifying the test parameters in order to cover the pathophysiological variability that the product could face in patients. In this study, we conclude that two, out of three marketed products, namely Foster^®^ NEXThaler^®^ and Seretide^®^ Diskus^®^, are substantially not affected *in vitro* by flow rate through the inhaler in terms of both delivered dose and fine particle mass.

As far as the extrafine particle fraction (<2 μm) at 4 kPa pressure drop (60 L/min flow rate) is concerned, Foster^®^ NEXThaler^®^ and Symbicort Turbohaler^®^ are capable of generating 50% and 30%, respectively, of this particle population over the total delivered dose. This would result in a reduced oropharyngeal drug deposition due to the low inertial impaction of the extra fine particles.

Furthermore, the effect of different inhalation volumes, tested only on Foster^®^ NEXThaler^®^, demonstrated that the performance of this product is unaffected by such a parameter. This positive characteristic can be mainly attributable to the presence of a breath-actuated mechanism guaranteeing the dose is released only when a threshold inspiratory flow is achieved.
